# Practical Use of the Ophthalmometer1Read before the Buffalo Ophthalmological Club, January 12, 1905.

**Published:** 1905-03

**Authors:** Lorenzo Burrows

**Affiliations:** Buffalo, N. Y.; Attending Ophthalmologist, Buffalo General Hospital


### Practical Use of the Ophthalmometer.[1]

By LORENZO BURROWS, M. D., Buffalo, N. Y.
Attending Ophthalmologist, Buffalo General Hospital.

INASMUCH as most of the difficulties experienced in getting favorable results with the ophthalmometer arise from improper handling, I have undertaken to describe and give the reasons for my own method of operating it, which for a good many years has yielded as nearly perfect satisfaction as any one test can.

POSITION.

With the table and chin rest so adjusted that the patient can comfortably keep the head in position with the eyes at the height of the white marks on the sides of the frame, the chin is set firmly on its rest; then from behind, the operator grasps the patient's head and tips it forward, with the chin as a fulcrum, guiding with the fingers, so that when the forehead touches its rest, there will be the same space on either side, between the temples and the sides of the head frame; and the skin of the forehead will lie smoothly in its normal position and not drawn in any direction. The patient must maintain a steady pressure with both the chin and the forehead. This is the only way to get the head straight. Bringing the eyes to a level by sighting through the horizontal slit in the disc, as is usually advised, is entirely wrong, because, generally they are not at the same level in the head and tilting it sidewise to make them so, is not the natural way of carrying it; and if the reading is taken in that position, the axis will be at fault, unless the tilting is so slight that it can be overcome by tortion, and the fact that the axis as indicated by the instrument, after leveling the eyes, so often agrees with that indicated by the trial lenses, is a strong argument against the statement that there is no such movement as tortion; without it, the two findings would disagree whenever the patient's face was asymmetrical, that is to say, almost always.

FOCUSING.

When using the old instrument that tilts forward or backward, to bring the telescope in line with the eye, it is necessary to refocus it after the mires are moved to the secondary position; because the primary position being say, with the mires in the horizontal plane, and the whole instrument standing at an appreciable angle, with the top nearer the eye than the bottom; when the mires are rotated to the vertical plane, the upper one

1. Read before the Buffalo Ophthalmological Club, January 12, 1905.

will lie inside the original focus and the lower one outside, and an accurate reading will be impossible because of the resultant blurred images. The newer instruments, adjusted to raise and lower by a screw, leaving the disc in the same plane, should be focused in the primary position and not changed.

### READING.

If possible, the amount of overlapping must be read with the lids opened naturally; because their pressure upon the eyeball often makes a marked difference in the amount of astigmatism; sometimes as much as one diopter. This fact is used as the basis for the statement that the patient's eyes should be kept wide open in order to avoid an artificial showing; whether it is or is not artificial, it is a condition that is constant and the ciliary muscle has to meet it; therefore, it must be corrected as it stands and not as it theoretically should be.

### APPLICATION OF READING.

The general rule is, to substract from the reading a quarter or half diopter, when the astigmatism is with the rule, and add a quarter or half diopter when against the rule, the result representing the amount of correction the eye will accept. This is ordinarily applicable, but is subject to variations ranging from nothing up to one diopter and occasionally it will be found necessary to reverse the order and add when the astigmatism is with the rule, etc. Allowing for lenticular astigmatism, is the reason commonly given for making these reductions or additions; an asymmetry of the curvature of the posterior surface of the cornea and fundus astigmatism have also been suggested. Whatever may be the reason for it, the fact stands, that it must be done, and the point to keep in mind is that it must not be done arbitrarily; it is not less elastic than any other rule governing refraction. Some operators seem to disregard it entirely, and look upon the showing of the instrument as absolute which is a grievous mistake, because when the reading is less than one-half diopter with the rule, the astigmatism is nearly always against the rule, perhaps as much as one diopter.

### AXIS—PRIMARY AND SECONDARY POSITION.

In a large proportion of eyes, the meridians of greatest and least curvature are not exactly ninety degrees apart; that is, the astigmatism is irregular.

Roughly stated, about one-third of all cases show this irregularity in one or both eyes. The variation may be anywhere between five and forty degrees, and is most frequently found associated with astigmatism of less than one diopter, although quite an appreciable number may be noted with astigmatism of one to two diopters. A difference is rarely seen in the higher degrees.

In applying this part of the finding, it must be remembered that the axis indicated by the long pointer, when the mires are in the primary position, *i. e.*, at or near the horizontal plane,—is that for a minus cylinder if with the rule or a plus cylinder if against the rule; and the axis indicated by the long pointer when the mires are in the secondary position,—*i. e.*, at or near the vertical plane,—is that for a plus cylinder if with the rule or a minus cylinder if against the rule; this is because the axis indicated in the primary position is obtained by exactly aligning the mires in the horizontal meridian and this can be done only at the horizontal axis; and the axis indicated in the secondary position is obtained by exactly aligning the mires in the vertical meridian and this can be done only at the vertical axis. This interpretation of the finding is almost invariably correct.

### REASONS FOR DISAGREEMENT WITH TRIAL LENS FINDINGS.

*In amount of astigmatism found.*

1. Having the patient open the eyes unnaturally wide, because, pressure of the lids upon the eyeball increases astigmatism with the rule and decreases astigmatism against the rule. Drawing the skin of the forehead may add to or detract from this pressure.
2. Taking the reading with the instrument improperly focused, because the farther the telescope is from the eye the larger the images will be and the more they will overlap, and vice versa.
3. Failure to correctly apply the rule for reducing from or adding to the reading.
4. Latent astigmatism.

*As to the Axis:*

1. Arbitrarily assuming that the secondary position is ninety degrees from the primary instead of finding it independently—because one-third of the cases will show some irregularity.
2. Tilting the patient's head, to bring the eyes to the same level, because they are most always placed at different levels in the head.

482 FRANKLIN STREET.

---

A SANATORIUM for consumptives, intended as a memorial of King Humbert I., was opened at Leghorn on November 19, 1904, in the presence of the King of Italy.—*Med. Age.*

